# Age and Sex in the Development of Hepatic Encephalopathy: Role of Alcohol

**DOI:** 10.3390/biology13040228

**Published:** 2024-03-29

**Authors:** Xiao Y. Tong, Hussain Hussain, Nagarajarao Shamaladevi, Michael D. Norenberg, Aya Fadel, Omar El Hiba, El got Abdeljalil, Bilal El-Mansoury, Deepak Kempuraj, Sampath Natarajan, Andrew V. Schally, Miklos Jaszberenyi, Luis Salgueiro, Michael J. Paidas, Arumugam R. Jayakumar

**Affiliations:** 1 Department of Pathology, University of Miami Miller School of Medicine, Miami, FL 33136, USA; tng_xy@yahoo.com (X.Y.T.); mnorenbe@med.miami.edu (M.D.N.); 2 Department of Internal Medicine and Infectious Disease, Larkin Community Hospital, Miami, FL 33143, USA; hussainhussainmd77@gmail.com; 3 Molecular Analytics, Miami, FL 33187, USA; nithura7@yahoo.com; 4 General Medical Research, Neuropathology Section, R&D Service, Veterans Affairs Medical Center, Miami, FL 33125, USA; andrew.schally@va.gov (A.V.S.); cleofide1731@gmail.com (M.J.); luis.salgueirotosta@va.gov (L.S.); 5 South Florida VA Foundation for Research and Education Inc., Veterans Affairs Medical Center, Miami, FL 33125, USA; 6 Department of Internal Medicine, Ocean Medical Center-Hackensack Meridian Health, Brick, NJ 08724, USA; ayafadel167@yahoo.com; 7 Laboratory of Anthropogenic, Biotechnology, Health, and Nutritional Physiopathologies, Neuroscience and Toxicology Team, Faculty of Sciences, Chouaib Doukkali University, Av. Des Facultés, El Jadida 24000, Morocco; oelhiba@gmail.com (O.E.H.); elmansoury1995bilal@gmail.com (B.E.-M.); 8 Hassan First University of Settat, Higher Institute of Health Sciences, Laboratory of Sciences and Health Technologies, Epidemiology and Biomedical Unit, Settat 26000, Morocco; abdeljalil.elgot@uhp.ac.ma; 9 Department of Neurology, School of Medicine, University of Missouri, Columbia, MO 65211, USA; dkx2c@missouri.edu; 10 U.S. Department of Veterans Affairs, Harry S. Truman Memorial Veterans Hospital, Columbia, MO 65201, USA; 11 Department of Chemistry, School of Chemical and Biotechnology, SASTRA Deemed University, Thanjavur 613401, India; sams76@gmail.com; 12 Pathology, Laboratory Medicine, Endocrine, Polypeptide and Cancer Institute, Department of Veterans Affairs, Miami, FL 33125, USA; 13 Department of Pathophysiology, Faculty of Medicine, University of Szeged, 6720 Szeged, Hungary; 14 Department of Obstetrics, Gynecology & Reproductive Sciences, University of Miami Miller School of Medicine, Miami, FL 33136, USA; mxp1440@med.miami.edu; 15 Department of Biochemistry & Molecular Biology, University of Miami Miller School of Medicine, Miami, FL 33136, USA

**Keywords:** alcohol, azoxymethane, brain edema, hepatic encephalopathy, thioacetamide

## Abstract

**Simple Summary:**

Hepatic encephalopathy (HE) is a major neurological condition that occurs following acute or chronic liver failure. There are two types of HE: acute (Type A) and chronic (Type C). The acute form leads to rapid coma and death due to increased intracranial pressure, while neurobehavioral abnormalities potentially characterize the chronic form. There is currently no available treatment to regress or cure HE. This may be due to various factors, of which age and sex are the most important to consider when treating patients with HE. Accordingly, we examined whether sex and age play a role in the development of HE. We found that drug-induced (thioacetamide, TAA) brain edema was more severe in aged males than in young males or young/aged female rats. Furthermore, adding alcohol to the young male or young/aged female rats aggravated the brain edema. Regarding the behavioral changes, TAA-induced deficits were less pronounced in young and aged females than in aged males, whereas in the chronic type, young and aged males showed no behavioral changes when alcohol was infused along with TAA despite the severe brain edema and death that occurred. These findings suggest that aged males and young/aged females are more prone to developing brain edema in the presence of one or more liver toxins.

**Abstract:**

Hepatic encephalopathy (HE) is a neurological condition linked to liver failure. Acute HE (Type A) occurs with acute liver failure, while chronic HE (Type C) is tied to cirrhosis and portal hypertension. HE treatments lag due to gaps in understanding its development by gender and age. We studied how sex and age impact HE and its severity with combined liver toxins. Our findings indicate that drug-induced (thioacetamide, TAA) brain edema was more severe in aged males than in young males or young/aged female rats. However, adding alcohol (ethanol, EtOH) worsens TAA’s brain edema in both young and aged females, with females experiencing a more severe effect than males. These patterns also apply to Type A HE induced by azoxymethane (AZO) in mice. Similarly, TAA-induced behavioral deficits in Type C HE were milder in young and aged females than in males. Conversely, EtOH and TAA in young/aged males led to severe brain edema and fatality without noticeable behavioral changes. TAA metabolism was slower in aged males than in young or middle-aged rats. When TAA-treated aged male rats received EtOH, there was a slow and sustained plasma level of thioacetamide sulfoxide (TASO). This suggests that with EtOH, TAA-induced HE is more severe in aged males. TAA metabolism was similar in young, middle-aged, and aged female rats. However, with EtOH, young and aged females experience more severe drug-induced HE as compared to middle-aged adult rats. These findings strongly suggest that gender and age play a role in the severity of HE development and that the presence of one or more liver toxins may aggravate the severity of the disease progression.

## 1. Introduction

Hepatic encephalopathy (HE) is one of the major neurological disorders linked to mild to moderate, as well as severe, liver disease, which occurs in acute and chronic forms with a mortality rate of up to 50% [1,2,3,4]. Acute HE (Type A HE) occurs following massive liver necrosis due to viral hepatitis or exposure to various hepatotoxins [1,5,6,7,8,9]. Drug-induced Type A HE, often from acetaminophen overdose, is a significant concern, especially in cases of suicidal overdose and pregnancy-related poisoning [7]. Notably, the rate of overdose incidence is higher among females than in males [1,10,11]. This may be due to the greater severity of drug-induced Type A HE along with other complications (e.g., hepatitis) in females than in males, although there is no clear literature available to date in this regard. This study, therefore, investigates sex and age differences in the development of HE and whether the severity of HE can be aggravated in the presence of additional liver toxins.

Chronic liver failure (Type C HE) often arises from liver cirrhosis, leading to severe neuropsychiatric symptoms that impact patients’ lives significantly [12,13,14,15]. Approximately 5.5 million individuals in the USA suffer from Type C HE [12,15]. Decompensated cirrhosis is marked by entirely symptomatic overt HE (OHE), occurring in 30–40% of cases, while minimal HE (MHE) affects 20–80% of cirrhosis patients [14]. The US has seen a rise in HE cases, with related hospitalizations increasing by 33% in 2018 and the cost for each patient per week averaging USD 20,000 [16,17,18]. HE is also a major health concern all over the world.

Patients with Type C HE experience a range of neurological symptoms, including mood swings, sleep disturbances, muscle tone changes, and severe cognitive deficits [1,15,19,20]. Chronic liver disease (CLD), often linked to alcoholism, nonalcoholic steatohepatitis (NASH), or hepatitis B/C, is increasingly acknowledged for its impact on morbidity and mortality [19,20,21,22,23]. As CLD and cirrhosis become more prevalent, HE’s prevalence is expected to follow suit. Notably, Type C HE leads to irreversible brain dysfunction from liver failure, even if the underlying liver issue is treated successfully [19].

While the precise molecular basis for the neurological disorder associated with Types A and C HE remains elusive, the dominant view has been that the diseased liver does not adequately eliminate gut-derived ammonia. Ammonia then enters the systemic circulation and, ultimately, the brain, which exerts deleterious effects [24,25,26,27,28,29]. Blood, CSF, and brain ammonia levels are elevated in human and experimental HE, and such increases ultimately result in Type A HE [24,25,26,27,28,29]. In contrast to the involvement of ammonia in the development of Type A HE, clinical examination shows inconsistency between blood ammonia level and patient clinical outcomes. Some studies even emphasized that increased ammonia does not contribute to the disease progression [30,31]; instead, changes in gut microbiota are the evolving concept in the pathogenesis of Type C HE [19,21,22]. Recent studies have highlighted the gut microbiota’s potential role in the mechanism of Type C HE [21,22,32].

While both Types A and C HE can be developed due to liver damage by viral hepatitis or hepatotoxins such as acetaminophen [19,33,34,35], the differing contribution of these liver toxins to the development of these encephalopathies remains unclear. Interestingly, most Type C HE cases progress to Type A HE, and conventional treatments have not notably improved the outcomes. This may be due to limited mechanistic insights into the development of Types A and C HE and a lack of age and sex variation data. Most studies have focused on males, hindering our understanding of female-specific mechanisms. The lack of data on females and age differences impedes the applicability of results obtained from adult males to young, adult, or aged females. Relying on male animals in research has left gaps in data translation to clinical trials with both genders. Accordingly, we examined whether sex and age differences play a role in the development of HE and whether the severity of HE can be aggravated in the presence of one or more liver toxins.

## 2. Materials and Methods

### 2.1. Animals

Adult male Wistar rats weighing 180–200 g (Charles River Laboratories Inc., Wilmington, MA, USA) were maintained in a 12 h light/dark cycle and supplied with standard laboratory chow and water ad libitum. All animals were free of infection at the onset of the experiment. All procedures were carried out according to the guidelines established by the National Institutes of Health for animal care (Guide for the Care and Use of Laboratory Animals) and approved by our Institutional Animal Care and Use Committee (IACUC).

### 2.2. Type A HE in Rats

Thioacetamide (TAA)-induced acute HE in rats was used in this study [36,37,38,39,40,41,42,43,44]. The TAA-treated rat model of Type A HE has been well-established relative to the clinical status and liver and brain function [36,37,38,39,40,41,42,43,44]. It should be highlighted that this is the most widely used animal model to study acute HE. In this model, rats were given TAA (300 mg/kg body weight, i.p.) for 3 consecutive days. Control rats received normal saline only. To prevent hypoglycemia, rats were given 12.5 mL/kg body weight of an iso-osmolar solution (at 12 h intervals, s.c.) consisting of 5% dextrose and 0.45% saline with 20 mEq/L of potassium chloride [36,37,40,41,42,43,44]. The TAA rat model’s clinical status and liver function have been previously established [39,40,41,42,43,44]. Rats were clinically monitored at least twice a day and stages of encephalopathy were graded according to the criteria of Gammal et al. [45]: grade I: lethargy (generalized reduction in spontaneous activity); grade II: mild ataxia; grade III: lack of spontaneous movement but intact righting reflex; grade IV: loss of righting reflex but intact pain reflex (measured by the reaction to tail pinch); and grade V: coma [45]. Body temperature and weight were monitored in these animals, which were sacrificed 24 h after the last injection. TAA administration results in severe hepatocellular necrosis and increased blood and brain ammonia associated with the decline in neurological function and brain edema (enlarged, vacuolated nuclei and pale/expanded cytoplasm in the astrocytes of the cerebral cortex with the neuropil highly vacuolated (spongiotic), especially around blood vessels and neurons) [39,40,41,42,43,44].

### 2.3. Azoxymethane (AZO) Model of Type A HE

Azoxymethane (AZO)-induced acute liver failure in mice was also used in this study to evaluate better the impact of liver failure in a rat model with TAA. Briefly, AOM (Sigma-Aldrich, St. Louis, MO, USA) was dissolved in 100 μL saline and injected intraperitoneally (100 µg/g) as previously described [46]. Control animals received an equivalent volume of saline. Mice were given 5% dextrose in saline and maintained at 37 °C. This model produced hyperammonemia, cerebral edema, microglial activation, and an upregulation of neuroinflammatory mediators (characteristic features of acute HE) [46,47,48].

### 2.4. Type C HE in Rats

An established rat model of chronic liver failure was used in this study [41,44,49,50,51]. Briefly, chronic liver failure was induced in Wistar rats (Fisher-344) (160–175 g) following the injection of TAA (100 mg/kg b.wt. in 0.9% NaCl, i.p.) for 10 consecutive days. Control rats were given the same volume of 0.9% NaCl. To minimize weight loss, hypoglycemia, dehydration, and renal failure in TAA-treated rats, 5% dextrose containing 0.45% NaCl and 20 mEq/L of potassium chloride were added to the drinking water.

Previous reports of this model have described hepatic damage (increased blood levels of alanine aminotransferase and aspartate aminotransferase), histopathological changes in the liver, alterations in brain biochemical parameters (an increase in neuronal nitric oxide synthase, NADPH oxidase activity, over-activation of glucose-6-phosphate dehydrogenase, and a decline in phosphofructokinase-2), and behavioral abnormalities characteristic of Type C HE [41,44,49,50,51].

We further characterized this model and found liver morphological changes (i.e., liver sections from TAA-treated rats showed ballooning degeneration, hydropic changes, and the presence of eosinophilic bodies, affecting approximately 60–70% of the liver parenchyma, predominantly in periportal regions). We also found increased blood and brain ammonia levels (two- to three-fold) [50,51,52]. Ammonia levels in these rats’ blood and brain tissue were also comparable with those found in rats after portocaval anastomosis [26].

EtOH was infused orally (0.40% *w*/*v*) in male and female, young and aged rats as described previously [53,54,55] with or without TAA (300 mg/kg). At the end of treatment, brain edema was measured. In a separate group of animals, EtOH was given with or without TAA (100 mg/kg), and behavioral analysis was performed from Day 5 post-TAA and terminated at Day 10 post-TAA injection (the time required for chronic TAA effect).

We used young (5–7 weeks), mature adults (3–7 months), middle-aged (9–14 months), and older (12–24 months) rats, which correspond well with the human aging process: young (17–23 years), mature adult (20–30 years), middle-aged (38–47 years), and older (58 and above). While it is possible that rats at ages below 5–7 weeks may be affected by liver toxins, and it is well-known that pediatric liver injury is a major clinical issue [34,35], and at ages 17 and below is also known to be affected by various liver toxins and viral hepatitis, EtOH use in humans in this age group (ages 17 and below) is not a major issue. We did not identify any significant change in brain edema or behavioral deficits when 100 mg/kg TAA was infused in rats under 5–7 weeks old or 24 months old; we excluded this age group from the current study.

### 2.5. Brain Edema Measurement

Brain water content was measured using the gravimetric method [50,51,52,56], and the values are compared with the results obtained with the wet/dry weight method. Rats were decapitated, and approximately 10 mg of cerebral cortical tissues was placed in a bromobenzene–kerosine density gradient column. The column was calibrated with varying concentrations of K_2_SO_4_ to ensure a linear relation (r = 0.998) between the specific gravity of K_2_SO_4_ and the density gradient. The equilibration point was read 2 min after placing samples in the column. Values from a minimum of 15 samples per rat were averaged, and the conversion from specific gravity to brain water was performed as described previously [50,51,52,56]. Brain water content was determined by the wet/dry method. Approximately 10 mg of tissue (five pieces from each animal) of the cerebral cortex was dissected; the wet weights of tissue were determined; the tissue was dried overnight in an oven at 100 °C; and the dry weights were determined. The difference in wet/dry weights was expressed as percent water content.

### 2.6. Behavioral Analysis

Male/female Fisher-344 rats (young—5 weeks, mature adult—6 months, middle-aged—10 months, and aged—24 months) received TAA or a vehicle daily for 10 days. Rats were then examined for behavioral abnormalities.

(a) Rota-rod test

As previously described, a Rota-rod apparatus was used to determine motor coordination [27]. This apparatus consisted of a horizontal rod with a roughened surface moving on its axis at 10 rpm/min. The rationale of this test was that the animals whose motor coordination had deteriorated dropped off from the rod into a tray 10 cm below. A test period of 60 s was allowed for each animal, and the endurance time was determined by measuring the time between the rat’s placement on the moving rod and the moment it fell. An animal’s endurance time of 60 s or more was taken as a normal response. Any reduction in the endurance time was taken as a reflection of the deterioration of motor coordination.

(b) Morris water maze

The Morris water maze task was used to evaluate spatial learning/memory deficits as described previously [57]. In brief, a circular pool tank (210 cm in diameter) was filled with water in a suitably equipped room with a constant temperature of 22 °C with humidity. For spatial learning in the acquisition (unseen platform), the experimental rats were required to find the invisible platform using spatial cues. A translucent acrylic platform (10 cm in diameter) was placed in the center of the corner quadrant and submerged 1 cm below the water surface throughout the training period. The rats were subjected to 4 trials/day at 4 min intertrial intervals for 5 days. The starting position was randomly chosen and counterbalanced across all the groups for each trial. The experimental rats were gently released into the water by facing the tank wall at 4 different starting positions. These rats were allowed to swim for a maximum of 60 s or until they located the submerged translucent acrylic platform and were allowed to stand on the submerged platform for 20 s. Any experimental rats that did not find the platform were led to the platform by hand and allowed to stand on it for 20 s. The time taken for each experimental rat to reach the hidden submerged platform was used as the escape latency for its spatial learning score. The time spent on the swimming track in the maze was recorded using a Black/White Box camera (EverFocus, MODEQ150, Orange, CA, USA) connected to a computer equipped with a self-contained video digitizer and tracking software (Videomex-One, Columbus, OH, USA). Twenty-four hours after the training period, a probe test in which the hidden platform was removed was conducted to assess spatial memory retention. In the probe test, the experimental rats were released into the water from the opposite quadrant to the target quadrant and allowed to swim for 60 s. The time spent by each experimental rat in the target quadrant that previously hosted the hidden platform was recorded as a measure of spatial memory. At the end of the experiment, the data tabulated by the video-tracking software were exported to a spreadsheet compatible with “CVS” files (Microsoft Excel, 15.0.4551.1011, Microsoft Corporation, Redmond, WA, USA) for further analysis of the spatial learning and memory parameters.

### 2.7. Thioacetamide Sulfoxide (TASO) Quantification

Plasma and liver samples were collected from rats after euthanasia at various time points after the i.p. injection of TAA (300 mg/kg). Liver samples were homogenized in saline (1 mL saline/g), and the homogenate was centrifuged at 2100× *g* for 20 min. The supernatant was then analyzed for TASO (a TAA metabolite) concentration. TASO was quantified by a reverse-phase HPLC as described previously using 7% acetonitrile, 50 mM sodium sulfate, and 50 mM potassium phosphate buffer (pH 7.4) as the mobile phase [39,58]. A semi-permeable surface ODS column was used to separate the components at 1 mL/min. TASO was detected by UV absorption at 290 nm using a photodiode array detector. The retention times for TA and TASO were approximately 4.1 and 3 min, respectively.

### 2.8. Blood Ammonia Estimation

Blood ammonia was measured. Blood was collected at the end of the treatment time. After centrifugation, ammonia levels in the serum were measured using a commercially available ammonia assay kit, following the manufacturer’s protocol (Cat# AA0100–1, Sigma–Aldrich, St. Louis, MO, USA).

### 2.9. Liver Function Test and Histopathology

To assess the extent of liver failure, blood levels of aspartate aminotransferase (AST) and alanine aminotransferase (ALT) were determined 4 h after the last injection of TAA using a Cobes 0501 automatic analyzer (Roche Diagnostics, Indianapolis, IN, USA). We also determined the liver histopathology of control and TAA-treated rats (extent of necrosis/steatosis). For this purpose, small liver samples were fixed in 10% formalin, processed routinely for paraffin sections, and stained with hematoxylin and eosin.

### 2.10. Statistical Analysis

Five to nine animals were used per group. Data from all experiments were subjected to an analysis of variance followed by Tukey’s multiple comparison test. A value of *p* < 0.05 was considered significant. Error bars represent the mean ± S.E.

## 3. Results

### 3.1. The Effect of TAA on the Development of Brain Edema

Previous reports have described hepatic injury (increased blood levels of alanine aminotransferase/aspartate aminotransferase, as well as histopathological changes in the liver); alterations in brain biochemical parameters; as well as behavioral abnormalities characteristic of Type C HE [43,44,46,49,50,51,52,56,57]. Further, blood and brain cortical ammonia concentrations were significantly increased in TAA-treated rats (two- to three-fold) [27]. We found that the ammonia levels in these rats’ blood and brain were similar to those observed in rats subjected to portocaval anastomosis [26,27]. These levels were also comparable to the ammonia levels in the blood of humans affected by Type C HE.

**Figure 1:**
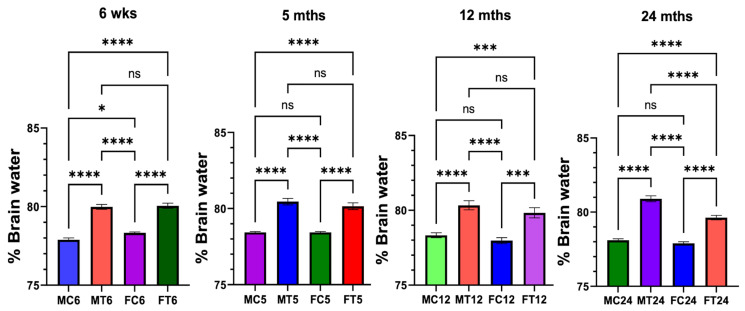
Drug-induced brain edema in male and female rats: Thioacetamide (TAA)-induced brain edema was more severe in aged male rats vs. young or mature adults or middle-aged rats. However, TAA-induced brain edema was not significantly different between young or mature adults and middle-aged or aged rats. *n* = 7 for controls and TAA. * *p* < 0.01, *** *p* < 0.0001, and **** *p* < 0.00001, ns = nonsignificant. Error bars represent the mean ± SEM. wks, weeks; mths, months. MC, male control; MT, male TAA; MA, male alcohol; MT, male TAA; MAT, male alcohol + TAA; FC, female control; FT, female TAA; FA, female alcohol; FAT, female alcohol + TAA.

We now show that TAA-induced brain edema was more severe in aged male rats vs. young, mature adult rats (Figure 1). However, there was no difference in the development of brain edema in any age group of female rats (Figure 1). Notably, the development of brain edema following treatment with TAA was significantly less severe in females than in males (Figure 1).

### 3.2. Effect of EtOH on Brain Edema

**Figure 2:**
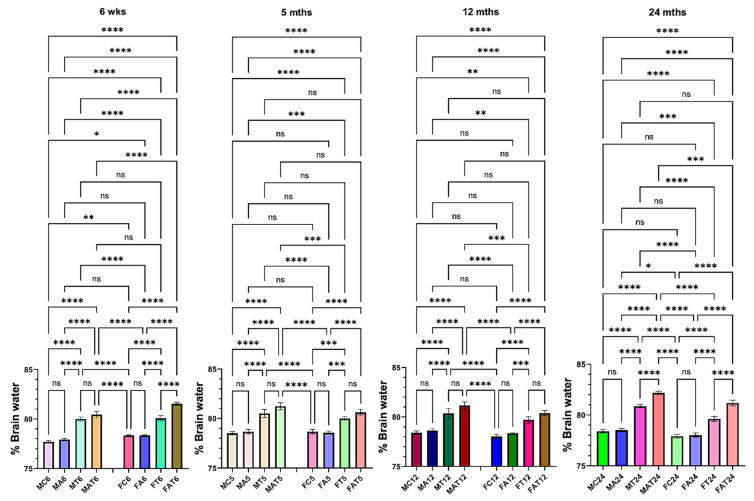
Effect of alcohol (EtOH) on drug-induced brain edema in male and female rats. TAA-induced brain edema was potentiated by alcohol in aged male rats. However, TAA-induced brain edema was potentiated by EtOH in both young and aged female rats. *n* = 7 for controls and TAA. * *p* < 0.01, ** *p* < 0.001 *** *p* < 0.0001, and **** *p* < 0.00001, ns = nonsignificant. Error bars represent the mean ± SEM. wks, weeks; mths, months. MC, male control; MT, male TAA; MA, male alcohol; MT, male TAA; MAT, male alcohol + TAA; FC, female control; FT, female TAA; FA, female alcohol; FAT, female alcohol + TAA.

The development of brain edema was exacerbated when aged male rats were exposed to EtOH along with the liver toxin TAA, as compared to young male rats (Figure 2). However, EtOH potentiated the effect of TAA on brain edema development in both young/aged females (Figure 2). Further, the effect of EtOH on TAA-induced brain edema was more severe in females than in males, although the extent of this increase was less than in males (Figure 2), suggesting that the more common Type A HE in women may be due to the presence of two or more liver toxins (e.g., EtOH, viral hepatitis, along with drugs). These findings strongly indicate that EtOH primarily determines the severity of brain edema in young vs. aged and in male vs. female rats.

#### 3.2.1. Effect of TAA +/− EtOH on Liver in Male

**Figure 3:**
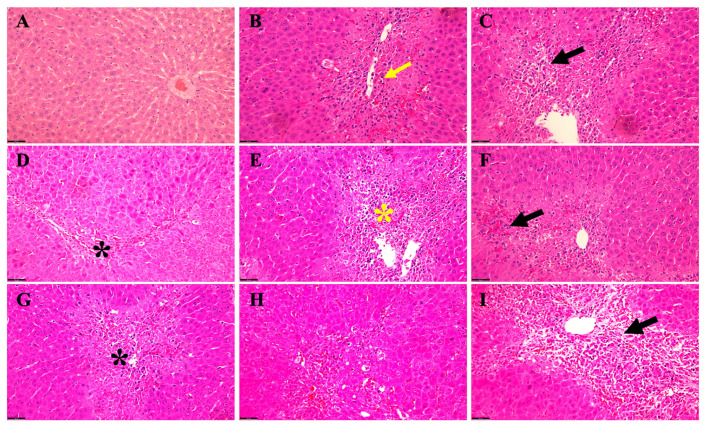
Acute hepatic histopathological changes in TAA alone versus TAA plus EtOH in a rat male model of HE. (**A**) A typical parenchymal liver architecture with a central vein. (**B**) (6 weeks TAA alone) Congestion and fatty changes (yellow arrow) in the cytoplasm of liver cells, hemosiderin disposition, and fibroblast cells. (**C**) (6 weeks TAA + EtOH) The severity of fat accumulation (black arrow) within hepatocytes with more hemosiderin scatters and fibroblastic cell proliferation, extending from the portal area to the hepatic parenchyma between the degenerated hepatocytes. (**D**) (5 months TAA) Fatty changes (black asterisk) in hepatocytes and mild congestion with some apoptotic hepatocytes. After adding alcohol (subfigure (**E**)) to those mice (5 months with TAA), they exhibited more fat, fibroblast accumulation, and congestion (yellow asterisk). (**F**) (12 months with TAA) We noticed mild–moderate fat and hemosiderin accumulation and congestion (black arrow) while adding alcohol to those rats, illustrating worsening hepatocyte changes (black asterisk) (**G**). The livers of rats treated with TAA alone at 24 months show fatty changes, fibroblast localization, apoptotic alteration, and congestion (**H**). In contrast, when alcohol was added to TAA in 24-month-old rats (**I**), the severity of fatty changes around the portal vein worsens with degenerative hepatocytes (black arrow), and more fibroblasts accumulate and increase the congestion level, as well as some fibrotic changes and inflammatory cells being recruited. Scale bar = 50 μm.

We identified various acute hepatic histopathological alterations induced by exposure to TAA alone compared to TAA in combination with EtOH (Figure 3). Microscopic evaluation revealed distinct changes in liver morphology across different time points and treatment conditions. Rats aged 6 weeks treated with TAA alone exhibited pronounced congestion and cytoplasmic fatty changes in hepatocytes, accompanied by hemosiderin deposition and fibroblast infiltration (Figure 3B). Concurrent administration of TAA and EtOH in these rats exacerbated hepatic lipid accumulation within hepatocytes, intensified hemosiderin deposition, and promoted fibroblast proliferation, notably extending from the portal triads into the hepatic parenchyma amidst degenerated hepatocytes (Figure 3C). In contrast, rats treated with TAA alone at 5 months of age displayed liver fatty changes and mild congestion, with sporadic occurrence of apoptotic hepatocytes (Figure 3D). Remarkably, adding EtOH to TAA in these rats exacerbated hepatic steatosis, fibroblast infiltration, and congestion (Figure 3E).

Rats aged 12 months exhibited mild to moderate hepatic lipid and hemosiderin accumulation alongside congestion when administered TAA alone (Figure 3F). Further, co-administration of EtOH with TAA at this stage exacerbated hepatocellular alterations (Figure 3G). Meanwhile, rats treated with TAA alone at 24 months of age displayed advanced hepatic changes, including prominent fatty changes, fibroblast infiltration, apoptotic alterations, and congestion (Figure 3H). Moreover, concomitant administration of EtOH and TAA at this stage exacerbated the severity of hepatic steatosis, particularly around the portal vein, accompanied by increased fibroblast infiltration, intensified congestion, worsening hepatocyte degeneration, and developing fibrotic changes (Figure 3I). These findings demonstrate the progressive nature of hepatic histopathological alterations induced by TAA exposure, which are further exacerbated by the co-administration of ethanol, particularly in aged male rats. This highlights the synergistic effect of TAA and EtOH in promoting hepatic injury and pathological changes associated with HE in this rat model.

#### 3.2.2. Effect of TAA +/− EtOH on Liver in Female

**Figure 4:**
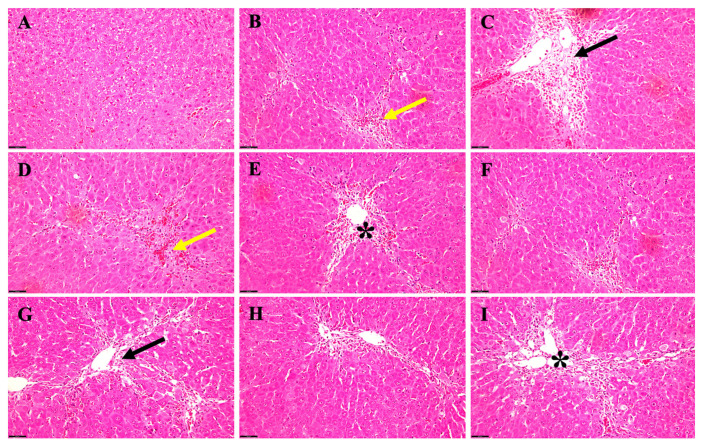
Acute histopathological changes in a rat female HE model induced by TAA alone versus TAA plus EtOH. (**A**) The normal parenchymal liver architecture. (**B**) A 6-week-old rat that received TAA alone; there is mild hepatocyte congestion (yellow arrow) and hemosiderin disposition with fibroblast cells. (**C**) A 6-week-old rat receiving both TAA + EtOH; there is a notable severity of the changes (black arrow), extending from the portal area to hepatic parenchyma between the degenerated hepatocytes. (**D**) Rats after 5 months of receiving TAA alone; the liver displays fatty changes and mild congestion (yellow arrows) with some apoptotic hepatocytes. However, adding alcohol (**E**) to those rats unveiled more steatosis (black asterisk) and fibroblast accumulation and worsened the congestion. There was mild–moderate fat and hemosiderin accumulation and congestion in (**F**) in 12-month-old rats, whereas adding alcohol to those rats illustrated worsening hepatocyte changes (black arrow) (**G**). The livers of rats treated with TAA alone at 24 months exhibited moderate steatosis, fibroblast localization, apoptotic alteration, and congestion (**H**). Furthermore, when EtOH was added to TAA in those rats (**I**), there was remarkable steatosis (black asterisk) around the portal vein with worsening hepatocyte degeneration, more fibroblast recruited, and an increased level of congestion, as well as some fibrotic changes and inflammatory cells being recruited. Scale bar = 50 μm.

In a rat female model of acute HE induced by TAA alone versus TAA in combination with EtOH, histological examinations were conducted to investigate the intricate alterations in liver architecture over time (Figure 4). During the early stages of the study, precisely at 6 weeks of age, rats subjected solely to TAA treatment (Figure 4B) exhibited observable yet mild manifestations, characterized by hepatocyte congestion and the presence of hemosiderin, along with the infiltration of fibroblast cells within the hepatic tissue. In stark contrast, their counterparts receiving TAA and EtOH (Figure 4C) displayed a notably heightened severity of hepatic changes. These alterations extended extensively from the portal area to the hepatic parenchyma, marked by the degeneration of hepatocytes (Figure 4C). As the study progressed to the 5-month mark, rats treated exclusively with TAA (Figure 4D) began to display signs of lipid accumulation within hepatocytes, accompanied by mild congestion and sporadic instances of apoptotic hepatocytes. However, when EtOH was introduced alongside TAA (Figure 4E), a clear worsening of hepatic steatosis, increased fibroblasts accumulation, and aggravated congestion became evident.

When rats reached 12 months, those exposed solely to TAA (Figure 4F) showed a progression in hepatic changes, with a moderate accumulation of fat and hemosiderin, alongside persistent congestion. Notably, adding EtOH to the treatment regimen (Figure 4G) further intensified the hepatocyte alterations observed, signifying a compounding effect of ethanol on hepatic pathology. In the later stages of the study, at 24 months, rats treated solely with TAA (Figure 4H) displayed a notable increase in hepatic steatosis, alongside the localization of fibroblasts, apoptotic changes in hepatocytes, and persistent congestion within the hepatic vasculature. Remarkably, incorporating EtOH into the TAA regimen (Figure 4I) led to a significant exacerbation of hepatic steatosis, particularly around the portal vein. Moreover, there was a notable aggravation in hepatocyte degeneration, increased recruitment of fibroblasts, heightened congestion, and the emergence of fibrotic changes, accompanied by a pronounced infiltration of inflammatory cells within the hepatic tissue.

While the histopathological alterations were observed in the acute hepatic encephalopathy (HE) model across both male and female rat cohorts, analogous variations were detected in the chronic manifestation of HE, albeit the specific data remain undisclosed.

### 3.3. Effect of Azoxymethane (AZO) on the Development of Brain Edema

**Figure 5:**
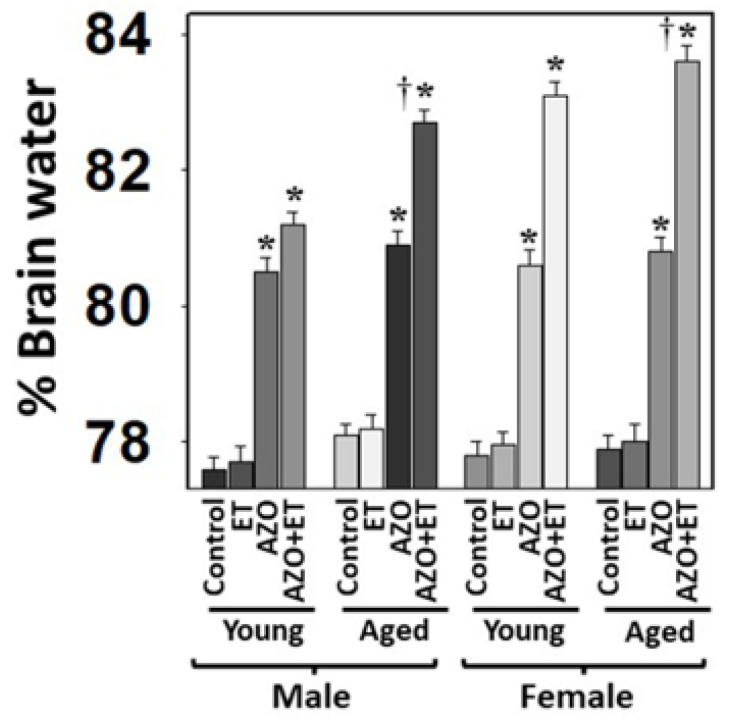
Effect of alcohol (EtOH) on drug-induced brain edema in mice. Azoxymethane (AZO)-induced brain edema was potentiated by EtOH (ET). ANOVA, *n* = 7 for controls and TAA. * *p* < 0.01 vs. control; † *p* < 0.01 vs. young rats. Error bars represent the mean ± SEM.

In addition to the rat model of TAA-induced HE, using more than one model of HE will increase our findings’ accuracy, viability, and translatability. Accordingly, we examined the role of age and gender in developing brain edema in the azoxymethane (AZO) model of Type A HE in mice [46,59] with and without EtOH. We found that brain edema in aged mice was more severe than in young mice (Figure 5). The brain edema was more severe in aged females when EtOH was added compared to aged males (Figure 5), while the young mice developed less severe brain edema when AZO was administered alone (Figure 5). These findings suggest that AZO is more toxic in the presence of EtOH and the findings with AZO plus or minus EtOH were similar to those observed with TAA and EtOH.

### 3.4. Effect of TAA on the Motor and Cognitive Functions

**Figure 6:**
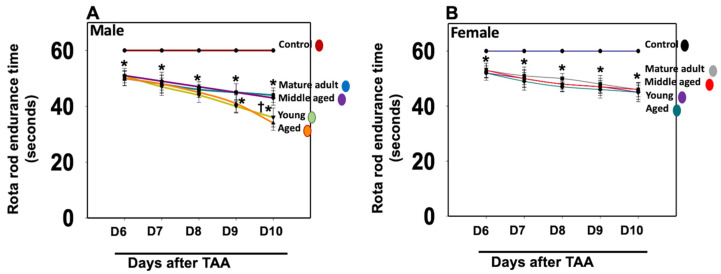
Motor coordination tasks in rats after TAA administration. Endurance time was shortened severely after the treatment of young and aged males with TAA compared to mature adult or middle-aged males (**A**). While endurance time was shortened after the injection of female rats with TAA, there was no difference between the age groups analyzed (**B**). Further, the reduction in endurance time in female rats was relatively less than that observed in males. ANOVA, *n* = 7 for controls and 9 for TAA. * *p* < 0.05 vs. control; † *p* < 0.05 vs. young and aged rats in males (**A**). Error bars represent the mean ± SEM. TAA, thioacetamide; D, days. The effects of TAA on spatial memory impairment were assessed using the water maze task. We found that the TAA-treated young/aged male rats exhibited impairments in acquiring the water-maze task compared to mature adults, middle-aged males, young/aged males, and adult female rats. Briefly, after 4 days of training, TAA-treated aged male rats showed a longer escape latency and path length to reach the platform (Figure 7) as compared to aged female rats (Figure 7). However, both young and aged male rats spent less time in the target quadrant (Figure 7) as compared to young and aged females (Figure 7). Animals that received TAA also showed a delayed match to location (working memory deficits). Together, these data document that TAA-treated young and aged male rats are more prone to neurobehavioral deficits than are young or aged female rats with Type C HE.

We assessed motor coordination and cognitive deficits in those rats to examine whether the development of neurobehavioral and cognitive deficits in Type C HE is age- and sex-dependent. We found the endurance time was markedly shortened after the treatment of young/aged males with TAA, as compared to mature adult or middle-aged males (Figure 6A). In contrast, the decrease in endurance time was less severe in females (Figure 6B). Further, the extent of endurance time reduction in Type C HE was far less in females than in males.

**Figure 7:**
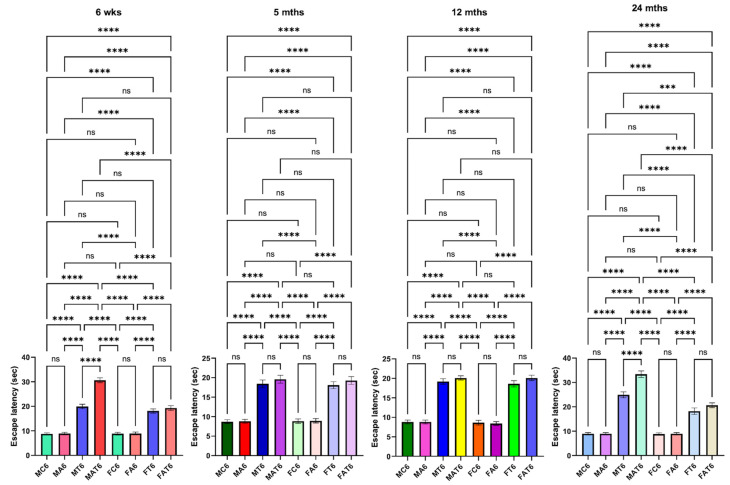
Spatial memory impairments in male and female rats with Type C HE, with and without EtOH (ET or Alco) exposure. Escape latency tests were performed for 4 days (between 4 and 10 days). TAA-treated young and aged male rats exhibited a longer escape latency and path length to reach the platform than young or aged female rats. ANOVA, *n* = 7 for controls and 11 for TAA. *** *p* < 0.0001, and **** *p* < 0.00001, ns = nonsignificant. Error bars represent the mean ± SEM. TAA, thioacetamide; wks, weeks; mths, months. MC, male control; MT, male TAA; MA, male alcohol; MT, male TAA; MAT, male alcohol + TAA; FC, female control; FT, female TAA; FA, female alcohol; FAT, female alcohol + TAA.

Additionally, behavioral abnormalities similar to those found in humans with Type C HE were observed in these rats, including drowsiness, decreased wakefulness and attentiveness, and impaired grooming and exploratory behavior. While animals with Type C HE were behaviorally defective, no evidence of severe distress, such as intense pain, muscular spasms, fainting, or breathing difficulties, was observed, consistent with features associated with Type C HE in humans, as well as with observations by other investigators in experimental animals [49,50,51,56,57,59,60,61]. These findings strongly suggest that young/aged male rats are more prone to develop TAA-induced Type C HE as compared to adult males or young or aged females.

### 3.5. Effect of EtOH on Behavioral and Cognitive Deficits

**Figure 8:**
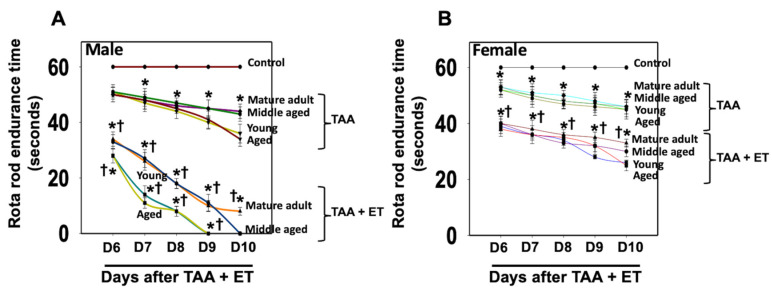
Motor coordination tasks in rats after TAA treatment with and without alcohol (ET). Endurance time was markedly reduced after treatment of young and aged males with TAA and TAA + EtOH (**A**) compared to mature adult or middle-aged males. While endurance time was shortened after exposure of female rats with TAA and TAA + EtOH (**B**), such a reduction was less than that observed in males. Further, drug-induced motor coordination deficit is more severe in all age groups in males (**A**) than in females after EtOH infusion (**B**). ANOVA, *n* = 7 for controls and TAA. * *p* < 0.05 vs. control; † *p* < 0.05 vs. TAA. Error bars represent the mean ± SEM. TAA, thioacetamide; D, days; ET, EtOH.

EtOH affects neurotransmitter systems, particularly gamma-aminobutyric acid (GABA) and glutamate, which can contribute to cognitive dysfunction and behavioral changes [62,63]. While we found that EtOH exacerbated the effect of TAA on brain edema in aged males and in young/aged females (Figure 2), drug-induced motor/coordination deficits in Type C HE were more severe in young/aged males (Figure 8A) than in young or aged females after EtOH infusion (Figure 8B). Notably, no behavioral changes were observed in aged males with Type C HE when EtOH was infused along with TAA; instead, severe brain edema and subsequent coma and death were identified (Figure 8A). These findings strongly suggest that EtOH contributes to the transition of Type C HE to Type A HE in a setting where drug-induced mild–moderate liver damage occurs. Further, both young/aged males are prone to chronic liver toxicity, and in the presence of EtOH, chronic liver damage evolves into fulminant hepatic failure.

**Figure 9:**
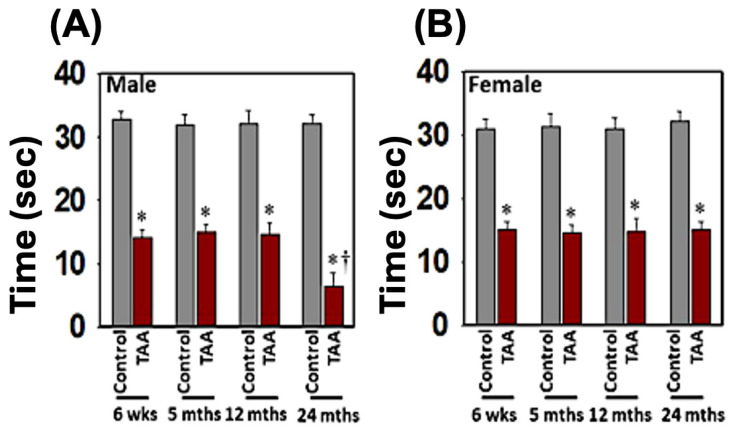
Spatial memory impairments in male (**A**) and female (**B**) rats with Type C HE. Time spent in the target quadrant during the probe trial indicates a deficit in spatial memory. ANOVA, *n* = 5 for controls and 7 for TAA. * *p* < 0.01; † *p* < 0.01. Error bars represent the mean ± SEM. TAA, thioacetamide; wks, weeks; mths, months.

The effects of EtOH on TAA-induced spatial memory impairment were also assessed using the water-maze task. Similar to the impact of EtOH on TAA-induced motor coordination imbalance, aged male rats exhibited severe impairments in the acquisition of the water-maze task, as compared to young, mature-adult, or middle-aged male rats when treated with TAA plus EtOH. Briefly, after 4 days of training, EtOH plus TAA-treated aged male rats showed a significantly longer escape latency and path length to reach the platform (Figure 7) as compared to controls or young, mature adult, middle-aged male, or female rats. EtOH/TAA-treated aged male rats also displayed significant deficits in escape latency during the acquisition of the water-maze task. These rats spent less time in the target quadrant, whereas control rats spent more time in the platform quadrant. Animals that received EtOH along with TAA also showed a delayed match to location (working memory deficits) (Figure 9). Some animals died at 8 to 9 days after treatment with EtOH and TAA. Altogether, these findings suggest that EtOH contributes to the transition of Type C HE to Type A HE in a setting where drug-induced mild to moderate liver failure occurs. Further, both young/aged male rats are prone to chronic liver failure (CLF), and in the presence of EtOH, the CLF ultimately evolves into Type A HE.

### 3.6. TAA Metabolism (Thioacetamide Sulfoxide, TASO Level)

Plasma and liver samples were collected from rats euthanized at various time points after TAA (300 mg/kg) i.p. injection. Liver samples (1 mL saline/g) were homogenized and then centrifuged. The supernatant was analyzed for TASO, a TAA metabolite, using a previously described reverse-phase HPLC assay [64,65,66]. A semi-permeable surface ODS column (5 µm; Regis Technologies, Morton Grove, IL, USA) separated components at 1 mL/min. Detection occurred at 290 nm using a photodiode array detector [64,65,66].

**Figure 10:**
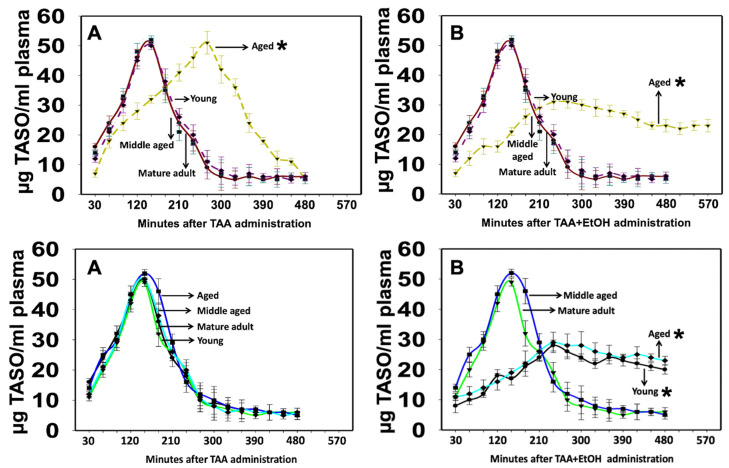
TASO content in plasma from TAA and EtOH-treated rats. (**A**) (Top panel) TAA was metabolized more slowly in aged males than in young or middle-aged rats. (**B**) (Top panel) A slow but sustained plasma level of TASO was observed when TAA-treated aged male rats were infused with EtOH, suggesting that the TAA-induced development of liver failure is more severe in the presence of EtOH. TASO content in plasma from TAA and EtOH-treated female rats. (**A**) (Bottom panel) TAA metabolism was similar in young or middle-aged, or aged female rats. However, a slow but sustained plasma level of TASO was observed when TAA-treated young and aged female rats were infused with EtOH (**B**) (Bottom panel), suggesting that, in the presence of EtOH, the drug-induced development of HE is more severe in young and aged females, as compared to middle-aged or mature adult rats. ANOVA, *n* = 5 for controls and TAA. * *p* < 0.05 vs. young, mature adult, and middle-aged rats in males ((**A**,**B**) top) and mature adult and middle-aged female rats ((**A**,**B**) bottom). Error bars represent the mean ± SEM. TAA, thioacetamide; EtOH, ethanol.

TASO was readily detected in plasma as early as 9 ± 4.1 min after the administration of TAA (300 mg/kg), indicating its rapid formation in both males and females of all ages (Figure 10). Plasma levels peaked at 120–150 min after the administration of TAA in all animals, except in aged males, where the peaks ranged from 250 to 290 min (Figure 10A, top panel). After reaching peak levels, TASO remained constant for 4–5 h in young and adult rats, while in aged males, the peak occurred at 5–6 h, and the TASO level remained constant for up to 7 h (Figure 10B, top panel) and declined rapidly after that. However, plasma TASO levels peaked at 120–150 min after administering TAA and quickly fell in all female rats (Figure 10A, bottom panel). In contrast, the administration of TAA to aged male and young and aged female rats with EtOH showed an increase in the TASO level at 120 min, which remained constant for up to 11 h (Figure 10B, top panel and Figure 10B, bottom panel) and then declined rapidly after that in both male and female rats. Further, the acetaldehyde (ACH) (a toxic EtOH metabolite) level was altered in TAA-treated aged male and young and aged female rats about TASO (i.e., TASO level decreased 1 h before the increase in ACH). In contrast, the ACH level decreased when the TASO level increased (data not shown).

These findings suggest that EtOH and TAA metabolites compete to inhibit their respective levels. Still, this event did not seem to involve a metabolite elimination issue since TASO was observed in the plasma for extended periods, indicative of a slower and more constant TAA metabolism into TASO, and subsequently to TASO2, which may have aggravated the severity of HE.

### 3.7. Acute Blood Ammonia Level and Liver Function Test

We found significant perturbations in blood ammonia levels and liver function parameters, including aspartate aminotransferase (AST) and alanine transaminase (ALT), as delineated in Table 1, Table 2, Table 3 and Table 4 among both male and female rats. Hence, similar changes were also observed in the chronic form of HE (data not shown).

## 4. Discussion

In this study, we found that exposure of male/female, young/aged rats to drugs alone will result in a significant increase in brain edema, and such edema development will be dependent on sex/age. Our findings suggest that the development of brain edema, mainly induced by toxins like TAA, is influenced by various factors such as age, gender, and the presence of ethanol. Further, brain edema was found to be more severe in aged males compared to young males or young/aged females when exposed to TAA. Interestingly, the effect of EtOH on TAA-induced brain edema was potentiated in both young and aged females, with females showing a more pronounced response than males. This pattern was consistent in a mouse model of AZO-induced Type A hepatic encephalopathy. Furthermore, behavioral deficits induced by TAA in Type C hepatic encephalopathy were less noticeable in females compared to aged males, while introducing EtOH alongside TAA had different effects on males and females, even resulting in severe fatality in males. Histopathological alterations were observed in the acute hepatic encephalopathy (HE) model across both male and female rat cohorts, concomitant with significant perturbations in blood ammonia levels and liver function parameters, including aspartate aminotransferase (AST) and alanine transaminase (ALT). Analogous variations were detected in the chronic manifestation of HE, albeit the specific data remain undisclosed. This underscores the complex interplay of gender, age, and toxins in the development of brain edema and associated conditions.

Thioacetamide induces liver injury free radical attack to macromolecules, leading to hepatocyte necrosis, particularly Zones 1 and 3, with more noticeable periportal injury than other toxins [6,36,37,38,39,58,59,64]. EtOH is one of the most common substances that potentiate liver injury through a complex mechanism. Acetaldehyde is a highly toxic product of ethanol to hepatocytes [67,68]. Further, ethanol triggers various reactive oxygen species (ROS), leading to severe liver inflammatory response and damage [68]. Acute liver failure leads to various neurological manifestations through a multifaceted mechanism, such as the development of brain edema and neurotoxicity.

The complex interplay of age and sex plays a pivotal role in shaping the response to the combined exposure of EtOH and TAA in brain edema development [69]. This interaction highlights the multifaceted nature of physiological and biochemical processes underlying brain edema susceptibility. Age-related variations in metabolic pathways, hormone levels, and cellular resilience can impact how the brain responds to the toxic insult of TAA exacerbated by EtOH [70]. Similarly, the distinct hormonal milieu and genetic factors present in male and female organisms can lead to differential outcomes in brain edema development under the influence of these compounds. The exploration of these age- and sex-dependent effects provides valuable insight into the nuanced mechanisms driving brain edema and emphasizes the importance of accounting for these factors in understanding and mitigating its pathological consequences.

A comparative investigation into the susceptibility of different age and gender groups of rats to brain edema, particularly in response to various hepatotoxic agents, yielded intriguing findings. Notably, in our study, the severity of brain edema differed significantly between aged male rats and their young, mature counterparts. Conversely, among female rats, no evident age-related differences were observed in the development of brain edema. Of particular interest is the impact of treatment with TAA. Remarkably, brain edema induction following TAA administration exhibited a notable sexual dimorphism. Female rats, in contrast to males, demonstrated a significantly attenuated tendency toward the development of brain edema upon exposure to TAA.

An intriguing facet of this study pertains to the interaction between TAA and EtOH. The concomitant administration of EtOH and TAA yielded distinct responses in different rat cohorts. Markedly, young male rats exhibited heightened susceptibility to exacerbated brain edema in the presence of both EtOH and TAA, a response notably accentuated in comparison to aged rats. The potentiating effect of EtOH on TAA-induced brain edema transcended gender barriers, as both young and aged female rats exhibited augmented susceptibility.

These findings suggest significant implications for our understanding of hepatic encephalopathy (HE) and its distinct phenotypes. Specifically, it is postulated that the differential prevalence of Type A and Type C HE in women could potentially be attributed to the presence of a combination of hepatotoxic agents [71,72]. Notably, the co-occurrence of agents such as EtOH and possibly infections alongside diverse pharmacological compounds may engender the development of HE in women [19,71,72]. Furthermore, the pivotal role of EtOH in influencing the severity of brain edema development emerges as a recurrent theme. In young versus aged rats, and in male versus female cohorts, EtOH prominently dictated the trajectory of brain edema. Intriguingly, a heightened sensitivity of female rats to the combined impact of EtOH and drug-induced Type A HE is proposed, suggesting a distinct susceptibility profile between genders.

Guy et al. reported that out of the 133 cases of drug-induced liver injury, a significant majority, 71%, occurred in women [72]. Furthermore, women comprised over 70% of all hospitalized patients experiencing acute liver injury due to acetaminophen and idiosyncratic drug reactions [72]. This higher prevalence of drug-induced liver injury in women may, in part, be attributed to gender differences in drug bioavailability, metabolism, and elimination and a higher rate of exposure to hepatotoxins, as extensively documented in both animal models (rats and mice) and humans [71,72,73,74]. Furthermore, the higher incidence of acute liver failure due to hepatotoxins does not have to affect the incidence of HE or its severity in women. Variations in the expression of cytochrome P450 enzymes play a crucial role in these differences, leading to varying susceptibility to drugs. For instance, women are more likely to express CYP3A4, a vital catalyst in liver oxidative metabolism and a key enzyme in the metabolism of many drugs [72,73,74].

The interplay between ethanol and thioacetamide in the context of Type C hepatic encephalopathy (HE) is a subject of significant scientific interest [73,74]. Ethanol’s impact on the liver’s metabolic functions, coupled with its potential to induce oxidative stress and inflammation, creates an environment conducive to hepatic impairment [75,76,77]. Thioacetamide, on the other hand, is a well-studied hepatotoxic agent often used in experimental models to induce liver damage, closely mirroring the pathological processes seen in chronic liver diseases [77]. When administered, TAA undergoes metabolic activation, generating reactive intermediates that contribute to liver injury through oxidative stress, inflammation, and cell death [78]. In our findings, brain edema developed when EtOH was given along with TAA in Type C HE (1.35–1.78%). Surprisingly, the death rate of animals with Type C HE in the presence of EtOH/TAA was higher. Although the reason for such a differential response of Type C HE animals to EtOH is unclear, the increased death rate could be due to defects in other organs (e.g., heart, kidney, lung), as multiorgan failure is a well-known cause of death in patients with HE.

In the context of Type C HE, the concurrent presence of both EtOH and TAA can result in a synergistic or additive effect on hepatic dysfunction, thereby potentially exacerbating the development of neurological symptoms and brain edema [74]. Ethanol’s impact on liver function may potentiate the hepatotoxicity induced by TAA, further compromising the liver’s metabolic and detoxification capabilities [74]. We noticed that in a combination of EtOH and TAA, distinct behavioral changes were observed in aged males in comparison to their younger counterparts. Further, there were no differences in behavioral/cognitive deficits in young vs. aged females. This interaction between age, EtOH, and TAA highlights the complexity of their combined influence on behavioral responses. The contrast in behavioral outcomes between aged and young males highlights the importance of considering age-related physiological variations when evaluating the effects of these compounds.

There are dissimilarities in the severity of hepatic encephalopathy (HE) in the acute phase between the two genders when exposed to two or more liver toxins [79,80,81]. The intricacies driving these variations remain elusive and necessitate further investigation. It is possible that there may be a tolerance which develops because the metabolism of the toxins speeds up between males and females (often because the liver enzymes involved in metabolizing toxins become more active and different between males and females) and because the number of sites (cell receptors) that the toxins attach to or the strength of the bond (affinity) between the receptor and drug decreases. Furthermore, there may be a delay in the metabolism of exogenous compounds into reactive intermediates in the liver when two or more toxins are present (e.g., the metabolism of TAA requires oxidative bioactivation, leading first to its S-oxide (TASO) and then to its chemically reactive S, S-dioxide (TASO2), that may be delayed due to the presence of EtOH, or that TAA may influence levels of the EtOH metabolite, acetaldehyde, a highly toxic substance which is known to cause liver injury), thus enhancing the bioavailability of the liver toxin for longer periods [81]. Accordingly, the toxicokinetic profile of a compound is critically important, especially when its toxicity depends on its bioactivation to a reactive intermediate in the presence of multiple compounds in the systemic circulation. Those toxins or factors may determine the severity of acute liver failure in males and females.

EtOH and various drugs compete for metabolism by CYP2E1, active drinkers often display enhanced sensitivity to certain drugs, as alcohol will inhibit the metabolism of the drug, thereby prolonging its half-life [81]. Conversely, since CYP2E1 is induced after chronic alcohol consumption, the metabolism of drugs which are also substrates for CYP2E1 will be increased [81]. This will decrease the half-life of the drug, thus decreasing the effectiveness of the drug when ethanol is not present. Furthermore, CYP2E1 is very active in oxidizing many chemicals into reactive intermediates, e.g., carbon tetrachloride, benzene, nitrosamines, acetaminophen, and halothane [81]. It is therefore possible that the toxicity of these agents may be enhanced in alcoholic patients. Advanced liver disease will decrease the rate of ethanol metabolism. Further, agents that inhibit alcohol dehydrogenase (ADH) (pyrazoles, isobutyramide) or that compete with ethanol for ADH (methanol, ethylene glycol) or inhibit the mitochondrial respiratory chain, will decrease the alcohol elimination rate. Antabuse (disulfiram), by inhibiting the elimination of acetaldehyde, slows alcohol metabolism, which may impact TAA/AZO metabolism and the subsequent availability of systemic and/or tissue TAA and EtOH metabolites for longer time periods [82]. This may result in a sustained liver injury that may subsequently aggravate the severity of HE.

The results presented indicate that women are less sensitive to motor and cognitive alterations in hepatic encephalopathy (HE) Type C. This finding is consistent with prior research suggesting sex-specific differences in the manifestation and severity of HE symptoms. For instance, a study by Norton et al. (1997) found that female rats with HE developed a shorter latency period in the stages of fulminant hepatic encephalopathy, as well as milder cognitive impairments compared to their male counterparts [83,84]. Furthermore, hormonal differences between sexes may contribute to variations in HE symptomatology. The exact reason is ambiguous, and further research is warranted.

While we found differences in developing brain edema and neurobehavioral deficits on a gendered basis in experimental models of liver failure, it is unclear whether this is what exactly happens in humans associated with HE. This may be a potential limitation when comparing animal studies with humans. Additionally, the factors that drive these differential responses to liver toxins may vary from humans associated with the disease progression. However, the signaling, if identified in the future, we strongly believe will provide useful information to nullify the disease progression in humans associated with HE, as other disease models were propagated.

## 5. Conclusions

Our study suggests that rats are more susceptible to drug-induced brain edema and are more sensitive to drug-induced brain edema when superimposed with two or more liver toxins. Drug-induced behavioral/cognitive deficits were severe only in males, and such effect was potentiated in the presence of other liver toxins (e.g., EtOH). These findings strongly suggest that a more targeted therapeutic approach based on the results obtained from our preclinical findings in young and aged males vs. females should be strongly considered in appropriate clinical settings.

## Figures and Tables

**Table 1:** Acute blood ammonia levels in male rats.

Acute HE (Blood Ammonia in Males)	6 Weeks Aged Rats	5 Months Aged Rats	12 Months Aged Rats	24 Months Aged Rats
Control	57.8 ± 14.9	61.8 ± 12.8	69.4 ± 18.4	89.2 ± 19.4
TAA alone	402.9 ± 28.6 *	476.9 ± 31.8 *	462.7 ± 28.6 *	502.7 ± 41.7 *
EtOH	59.2 ± 12.9	69.8 ± 11.9	72.8 ± 18.6	71.7 ± 21.6
TAA + EtOH	718.3 ± 101.8 †	616.7 ± 89.6 †	754 ± 121.7 †	818.4 ± 141.9 †

Mean values ± SD. Control *n* = 5; TAA *n* = 7; EtOH *n* = 5; TAA + EtOH *n* = 7. * *p* < 0.05 vs. control; † *p* < 0.05 vs. TAA. Hepatic encephalopathy (HE); alcohol (EtOH); thioacetamide (TAA).

**Table 2:** Acute blood ammonia levels in female rats.

Acute HE (Blood Ammonia in Females)	6 Weeks Aged Rats	5 Months Aged Rats	12 Months Aged Rats	24 Months Aged Rats
Control	44.6 ± 17.2	58.4 ± 11.9	64.8 ± 18.2	76.9 ± 14.9
TAA alone	364.6 ± 29.1 *	392.5 ± 31.8 *	418.1 ± 31.0 *	456.6 ± 28.6 *
EtOH	52.8 ± 15.7	61.7 ± 18.2	69.8 ± 21.6	70.6 ± 16.1
TAA + EtOH	802.4 ± 108.6 †	719.6 ± 126.2 †	724.2 ± 100.4 †	781.9 ± 92.8 †

Mean values ± SD. Control *n* = 5; TAA *n* = 7; EtOH *n* = 5; TAA + EtOH *n* = 7. * *p* < 0.05 vs. control; † *p* < 0.05 vs. TAA. Hepatic encephalopathy (HE); alcohol (EtOH); thioacetamide (TAA).

**Table 3:** Acute liver function test in male rats.

Acute HE (Liver Function Test, Male)	6 Weeks Aged Rats	5 Months Aged Rats	12 Months Aged Rats	24 Months Aged Rats
Control	ALT—49.2 ± 11.4 AST—97.9 ± 14.6	ALT—56.84 ± 9.68 AST—126.9 ± 18.6	ALT—62.4 ± 10.9 AST—131.7 ± 21.6	ALT—61.7 ± 16.4 AST—129.6 ± 19.2
TAA alone	ALT—816.9 ± 108.4 * AST—1987 ± 216.3 *	ALT—978.1 ± 116.8 * AST—2493 ± 316.9 *	ALT—959.3 ± 134.8 * AST—2652.7 ± 249.6 *	ALT—1162.9 ± 201.6 * AST—3108.9 ± 319.2 *
EtOH	ALT—69.3 ± 12.9 AST—141.3 ± 22.7	ALT—71.62 ± 14.9 AST—154.0 ± 21.6	ALT—81.9 ± 18.6 AST—168.7 ± 28.7	ALT—79.8 ± 20.3 AST—189.5 ± 28.6
TAA + EtOH	ALT—1219.6 ± 172.1 † AST—2801.6 ± 118.2 †	ALT—1311.7 ± 172.1 † AST—2819.6 ± 203.8 †	ALT—1272.8 ± 161.6 † AST—2918.3 ± 201.8 †	ALT—1619.4 ± 301.8 † AST—3672.1 ± 216.8 †≠

Mean values ± SD. Control *n* = 5; TAA *n* = 7; EtOH *n* = 5; TAA + EtOH *n* = 7. * *p* < 0.05 vs. control; † *p* < 0.05 vs. TAA; ≠ *p* < 0.05 vs. 6 weeks, 5 and 12 months. Hepatic encephalopathy (HE); alcohol (EtOH); thioacetamide (TAA), alanine aminotransferase (ALT); aspartate aminotransferase (AST).

**Table 4:** Acute liver function test in female rats.

Acute HE (Liver Function Test, Female)	6 Weeks Aged Rats	5 Months Aged Rats	12 Months Aged Rats	24 Months Aged Rats
Control	ALT—52.4 ± 10.6 AST—112.8 ± 20.6	ALT—46.3 ± 9.4 AST—83.7 ± 16.2	ALT—61.7 ± 11.8 AST—124.2 ± 21.3	ALT—61.9 ± 19.4 AST—122.8 ± 31.6
TAA alone	ALT—906.3 ± 82.1 * AST—2110.6 ± 186.2 *	ALT—800.9 ± 107.3 * AST—1914.6 ± 184.6 *	ALT—946.2 ± 119.1 * AST—2496.3 ± 206.8 *	ALT—1174.9 ± 126.8 * AST—2392.6 ± 201.8 *
EtOH	ALT—69.2 ± 14.8 AST—131.9 ± 18.6	ALT—56.9 ± 9.2 AST—124.2 ± 31.9	ALT—72.6 ± 12.8 AST—146.3 ± 21.8	ALT—76.4 ± 18.1 AST—157.5 ± 22.9
TAA + EtOH	ALT—1519.8 ± 154.2 †≠ AST—3514.7 ± 202.4 †≠	ALT—1138.4 ± 105.1 †≠ AST—2591.3 ± 200.3 †≠	ALT—1201.7 ± 94.2 †≠ AST—2617.4 ± 192.2 †≠	ALT—1612.4 ± 141.8 †≠ AST—3491.5 ± 246.8 †≠

Mean values ± SD. Control *n* = 5; TAA *n* = 7; EtOH *n* = 5; TAA + EtOH *n* = 7. * *p* < 0.05 vs. control; † *p* < 0.05 vs. TAA; ≠ *p* < 0.05 vs. 5 and 12 months. Hepatic encephalopathy (HE); alcohol (EtOH); thioacetamide (TAA); alanine aminotransferase (ALT); aspartate aminotransferase (AST).

## Data Availability

The data presented in this study are available on request from the corresponding author. The data are not publicly available due to the Miami VA privacy policy.

## Abbreviations

Hepatic encephalopathy (HE); acute HE (Type A); chronic HE (Type C); thioacetamide (TAA); alcohol (EtOH); azoxymethane (AZO); thioacetamide sulfoxide (TASO); overt HE (OHE); minimal HE (MHE); chronic liver disease (CLD); nonalcoholic steatohepatitis (NASH); gamma-aminobutyric acid (GABA); chronic liver failure (CLF); acetaldehyde (ACH); alanine aminotransferase (ALT); aspartate aminotransferase (AST); reactive oxygen species (ROS); chronic liver failure (CLF).
